# Role and Impact of a Clinical Pharmacy Team at an Inflammatory Bowel Disease Center

**DOI:** 10.1093/crocol/otad018

**Published:** 2023-04-15

**Authors:** David K Choi, David T Rubin, Archariya Puangampai, Monika Lach

**Affiliations:** University of Chicago Medicine, Inflammatory Bowel Disease Center, Chicago, Illinois, USA; University of Chicago Medicine, Department of Pharmacy, Chicago, Illinois, USA; University of Chicago Medicine, Inflammatory Bowel Disease Center, Chicago, Illinois, USA; University of Chicago Medicine, Inflammatory Bowel Disease Center, Chicago, Illinois, USA; University of Chicago Medicine, Department of Pharmacy, Chicago, Illinois, USA; University of Chicago Medicine, Department of Pharmacy, Chicago, Illinois, USA

**Keywords:** gastroenterology, clinical pharmacist, specialty pharmacy, access, affordability, advanced therapies, inflammatory bowel disease, biologics

## Abstract

**Background:**

There is limited literature describing the role of a clinical pharmacy team within a tertiary academic inflammatory bowel disease (IBD) center. The goal of this paper is to describe and showcase the clinical and operational impact of an integrated clinical pharmacy team.

**Methods:**

This was a retrospective study evaluating the referral outcomes for all patients referred to University of Chicago Medicine Specialty Pharmacy for self-administered advanced IBD therapies covered by prescription insurance from October 1, 2020 to October 31, 2021.

**Results:**

A total of 1800 referrals were received for advanced IBD therapies. Prior authorizations (PAs) were required and submitted for 1700 referrals. Of those 1700 PA submissions, 297 (17%) were denied by insurance. To overturn the denials, 344 appeals, including second-level appeals and external reviews, were submitted. Manufacturer patient assistance programs were obtained for 69 patients. From the 1800 referrals, 98% of patients were successfully started on the intended therapy. Clinically, there were 2141 pharmacist-initiated interventions by 2 IBD pharmacists. The most common interventions were prevention in interruption of therapy and providing patient education.

**Conclusions:**

Clinical pharmacy teams are well positioned to streamline care within a tertiary academic IBD center. Their unique skillset and ability to provide high yield medication access supports the use of this model as a best practice in IBD centers.

## Graphical Abstract

**Graphical Abstract:**
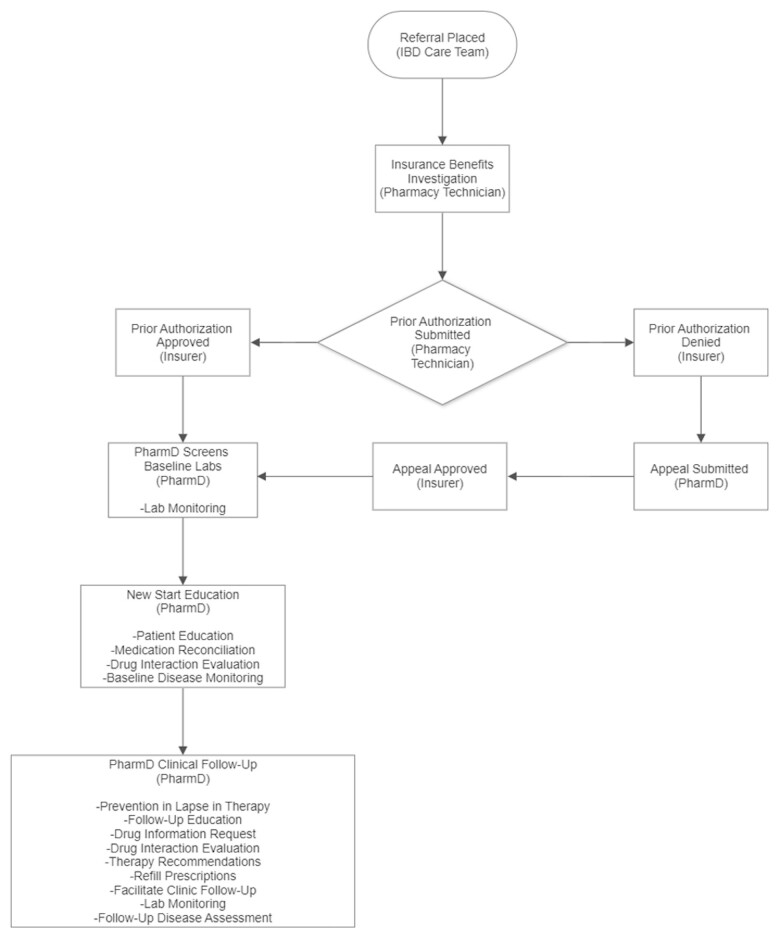

## Introduction

The inflammatory bowel diseases (IBD) are inflammatory conditions that include ulcerative colitis (UC) and Crohn’s disease (CD) and affect an estimated 3 million people in the United States.^1–3^ Advanced IBD therapies, including biologics and synthetic targeted small molecules, have changed the management strategies for IBD patients. These medications are expensive and require pre-authorization from third-party payers and require adequate and frequent monitoring to maximize efficacy and prevent complications associated with them.^1,2,4,5^

Given the increased complexity of care, there has been a growing emphasis on the utility of IBD medical homes to improve outcomes and care in this patient population.^6–8^ IBD medical homes have been shown to reduce emergency room visits, hospitalizations as well as improve disease activity and quality of life.^6–8^ Much like other areas in healthcare, there has been a push for value-based healthcare models for the IBD population by showcasing a decrease in healthcare utilization while improving disease control. Although IBD medical homes advocate for multidisciplinary care, pharmacists have not been acknowledged as integral members of the IBD medical home. However, outside of the IBD medical home model, pharmacists have demonstrated the impact they can make in improving safe and effective medication use.^9^

With the growth of advanced IBD-related therapies in the near future, 15 drugs currently in phase II/III trials for the treatment of IBD, there will likely be an increased need for clinical pharmacy teams to support and optimize therapies in these patients.^10^ It will be important to better understand the processes and impact that clinical pharmacy teams have on patient care and the IBD care team. The objective of this study is to identify the role of a clinical pharmacy team comprised of IBD pharmacists and a team of pharmacy technicians at a tertiary academic IBD center and to evaluate the operational and clinical contributions to patient care.

## Methods

### Study Design

This was a retrospective chart review performed at the Unviersity of Chicago Medicine from October 1, 2020 to October 31, 2021. All patients referred to the Unviersity of Chicago Medicine Specialty Pharmacy for advanced IBD therapies covered by prescription benefits were included, infusion therapies were not included as part of this study. Duplicate referrals were excluded. Data were collected using TherigySTM case management software (Therigy, LLC). Data collection included age, sex, IBD type, insurance type, advanced IBD therapy requested, reason for referral (ie, new therapy, dose adjustment, or continuation of therapy), date referral sent, date referral completed, prior authorization (PA) status, appeal status, and financial assistance enrollment status. Pharmacist-initiated clinical interventions by clinic embedded IBD pharmacists were also collected and categorized by type of intervention. This project received a formal Determination of Quality Improvement status according to Unviersity of Chicago Medicine institutional policy. As such, this initiative did not require review by the Institutional Review Board.

### Study Outcomes

The primary outcome of this study was to evaluate the total number of patients properly initiated on the provider requested advanced IBD therapy. Secondary outcomes were to assess the clinical interventions made by IBD pharmacists in relation to preventing lapses in therapy, providing patient education, addressing side effects, completing medication reconciliations, performing routine calls to assess adherence, offering recommendations to change therapy, dose adjust, or hold therapy, and coordinating care with follow-up visit recommendations.

### Referral Process

**Figure 1.**
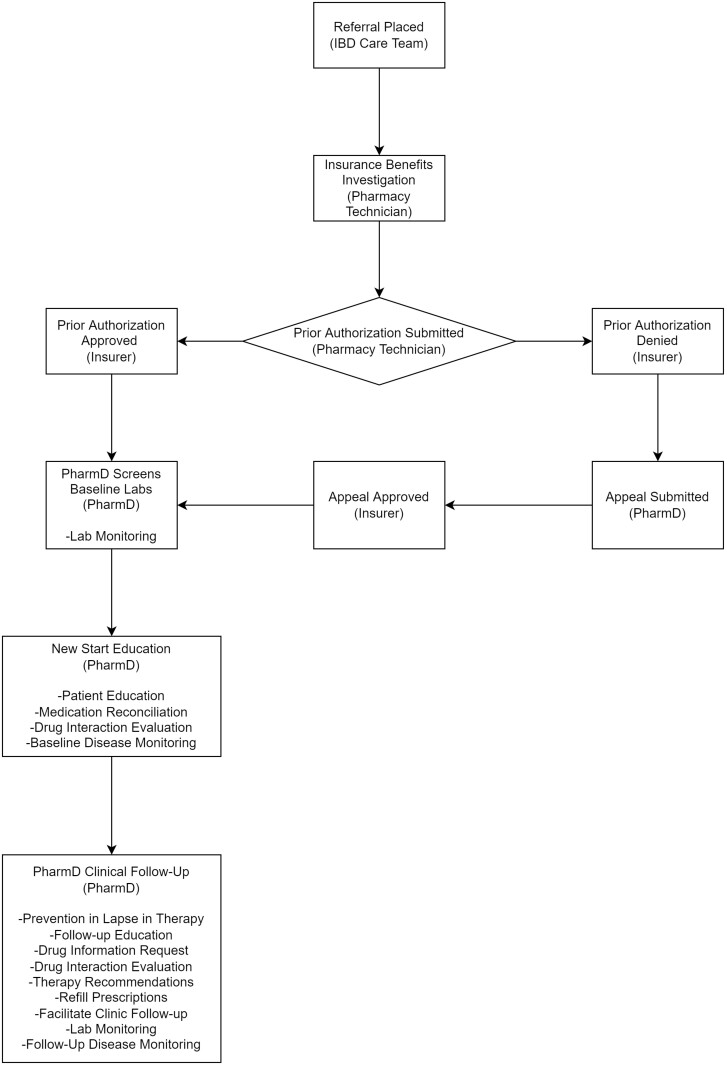
Framework for a clinical pharmacy team.

An IBD provider places a referral order in the electronic medical record to pharmacy to inform them of a start, change, or continuation of advanced IBD therapy (Figure 1). The referral is routed to a centralized order queue where a pharmacy technician initiates benefits investigation and submits the PA to the insurance. If the medication is not approved immediately by insurance, the pharmacy technician will follow-up for a status update 24–48 hours post submission. If the medication receives approval, the pharmacist e-prescribes the medication to the appropriate dispensing pharmacy, contacts the patient to start therapy and completes medication education. If the medication is denied, the pharmacist will immediately initiate an appeal to overturn the denial. Once the appeal is submitted, the pharmacy technician will follow-up for a status update 7–14 days post submission. If the appeal is approved, the same process is used as described above by the pharmacist. If the appeal is denied, the pharmacist will submit a second-level appeal if insurance permitting. If the second-level appeal is denied, an external review request will be submitted if insurance permitting. If the external review request is denied, the patient has no insurance or the medication co-pay is unaffordable, a dedicated financial assistance pharmacy technician will screen the patient to see if they are eligible for a patient assistance program and submit the application alongside the patient.

### Clinical Pharmacy Services

Pharmacists are currently embedded into clinics and provide patient care alongside the IBD care team (Figure 1). A clinical practice agreement is currently in place that allows the pharmacist to provide patient education, perform medication reconciliation, order labs, and send prescriptions for advanced therapies on behalf of providers. Patient education is provided on all new advanced IBD therapy starts. During the initial education, a medication reconciliation is completed, potential side effects are discussed and the patient is educated how to administer and store the medication. This education is developed in collaboration with the IBD center. Furthermore, there is a 24/7 pharmacist on call service that allows patients to directly contact a pharmacist to discuss side effects, drug interactions, and all other issues with their medications.

### Statistical Analysis

Shapiro–Wilk test was used to test normality. For descriptive statistics, median and interquartile range (IQR) was reported for nonparametric data, mean and SD was reported for parametric data. STATA v 13.0 was used for data analysis.

## Results

### Operational Interventions

From October 1, 2020 to October 31, 2021, 1800 patient referrals were sent to the clinical pharmacy team. Table 1 summarizes demographic characteristics of the referrals. The majority of patients had commercial insurance (86%) followed by Medicare (7%), then Medicaid (6%). Twenty-four patients (1%) had no prescription insurance at the time of referral. From the 1800 referrals, 1178 (65%) referrals were for continuation of therapy, 395 (22%) referrals were to start new therapy, and 220 (12%) referrals were dose adjustments of current therapy. The median days to referral completion was 11 days (IQR 6–20 days).

**Table 1.** Demographic results.

	Total (*n* = 1800)
Age, median (IQR)	36 (27–50)
Gender (% female)	996 (55)
IBD type	
Crohn’s disease (%)	1339 (74.4)
Ulcerative colitis (%)	440 (24.4)
Other (%)	21 (1.2)
Insurance type	
Commercial (%)	1544 (86)
Medicare (%)	125 (7)
Medicaid (%)	107 (6)
Uninsured (%)	24 (1)
Referral reason	
New therapy (%)	395 (22)
Continuation of therapy (%)	1178 (65.4)
Dose adjustment (%)	220 (12.2)
Days to outcome (IQR)	11 (6–20)
Referral for medication name	
Ustekinumab (%)	787 (44)
Adalimumab (%)	704 (39)
Tofacitinib (%)	186 (10)
Golimumab (%)	34 (2)
Certolizumab (%)	28 (2)
Ozanimod (%)	28 (2)
Upadacitinib (%)	25 (1)
Risankizumab-rzaa (%)	5 (<1%)
Apremilast (%)	2 (<1%)
Guselkumab (%)	1 (<1%)

Abbreviations: IBD, inflammatory bowel disease; IQR, interquartile range.

Of the 1800 referrals, 1700 (94%) required a PA submission while 100 referrals did not. Table 2 shows the number of patients properly initiated on provider requested advanced IBD therapy as well as approval rates. From the 1700 PA submitted, 1403 (83%) were approved upon initial submission. First-level appeals were submitted for 274 denials, and of those 209 denials (77%) were subsequently approved. Second-level appeals were submitted for 55 patients of those 23 denials (42%) were subsequently approved. At last attempt, 15 external review requests were submitted of those 11 (73%) were approved. Sixty-nine patients were successfully enrolled into a manufacturer patient assistance program. Twenty patients (29%) were enrolled due to being unable to obtain insurance approval and 49 patients (71%) were enrolled due to an unaffordable co-pay. In the end, a total of 34 patients referred (2%) were unable to start the intended therapy out of 1800 referrals and had to switch to an alternative therapy. Overall, approval for all medications was 76%–100%. In terms of medications that are approved by the Food and Drug Administration (FDA), there were 787 referrals for ustekinumab (approval rate of 97%), 704 referrals for adalimumab (approval rate of 99%), 186 referrals for tofacitinib (approval rate of 96%), 34 referrals for golimumab (approval rate of 94%), 28 referrals for certolizumab (approval rate of 100%), and 28 referrals for ozanimod (approval rate of 89%).

**Table 2.** Advanced IBD therapy access.

	Primary outcome (total 1800 referrals) *n* (%)	Approval rate %
No prior authorization required	100 (6)	NA
Prior authorization approved	1403 (78)	83
First-level appeal approved	209 (12)	77
Second-level appeal approved	23 (1)	43
External request appeal approved	11 (<1%)	77
Manufacturer patient assistance program	69 (4)	100
Unable to obtain insurance approval	20 (1)	
Unaffordable co-pay	49 (3)	
Unable to obtain approval	39 (2)	NA

Abbreviation: IBD, inflammatory bowel disease.

Referrals considered non-FDA labeled (ie, dose adjustments above FDA-labeled dosing and non-FDA-approved indications) accounted for 249 of the referrals (14%). The approval rate for non-FDA-labeled referrals was 92% (*n* = 228). At the time of this analysis, upadacitinib and risankizumab-rzaa were non-FDA-labeled therapies; however, these have now received approval for UC and CD, respectively. Apremilast and guselkumab are still considered non-FDA-labeled therapies for IBD.

### Clinical Interventions

**Figure 2.**
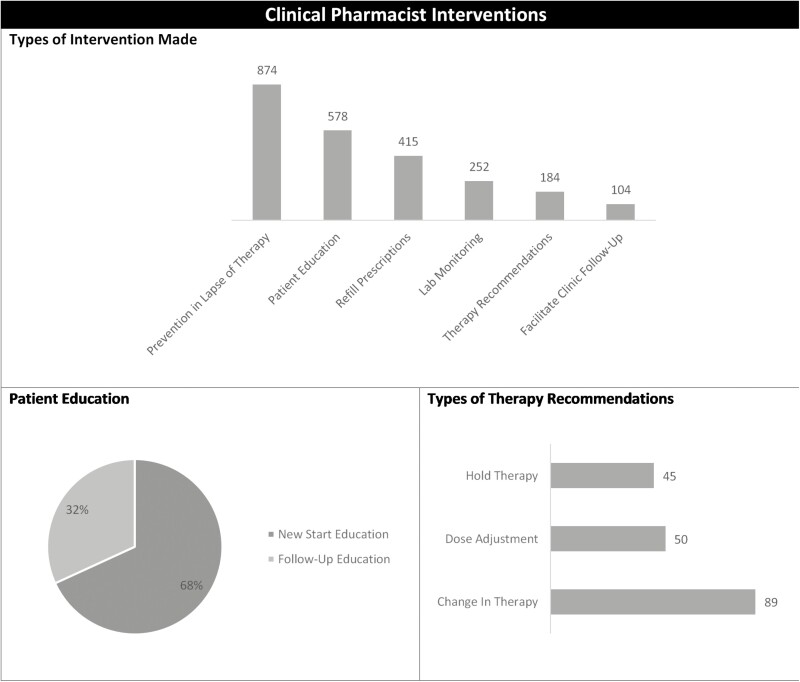
Clinical interventions.

From October 1, 2020 to October 31, 2021, a total of 2141 clinical interventions were documented by 2 IBD pharmacists (Figure 2). Of these clinical interventions, the predominant intervention was prevention in lapse of therapy which included phone calls made to outside specialty pharmacies on the patient’s behalf to troubleshoot issues as well as providing patients interim medication supply to prevent missed doses due to insurance approval barriers. The second most common clinical intervention was patient education. From the 578 education interventions, 395 were for new therapy initiations and the remaining 183 interventions were related to side effect management, drug interaction assessment, drug information questions, and adherence education. Clinical pharmacists facilitated refill prescriptions on behalf of the provider to outside specialty pharmacies decreasing clinic workload. Baseline lab recommendations were made in 252 patients prior to prescribing therapy to ensure the safe and effective use of these medications. Additionally, pharmacists were able were proactive in offering to change therapy, dose adjust, or hold therapy. With pharmacists closely monitoring these patients, a large number of clinic follow-up visit recommendations were made for patients to come in for a follow-up visit to ensure appropriateness of therapy and monitoring.

## Discussion

This is one of the first studies to comprehensively evaluate the impact of an IBD clinical pharmacy team at a tertiary academic IBD center. We demonstrate the importance and value of a clinical pharmacy team that is embedded as part of the IBD care team to ensure the safe, effective, and timely initiation of advanced IBD therapies.

Because IBD treatment is expensive, patients often experience insurance barriers that can lead to major delays in therapy initiation. It is advocated that a clinical pharmacy team, including IBD pharmacists and pharmacy technicians, be embedded in an IBD center to navigate these processes.^11^ As medication experts and given the understanding of the pharmacy benefit adjudication process, a clinical pharmacy team can ensure patient access to therapy.

A previous study assessing pharmacists practicing in gastroenterology and hepatology clinic found that 23% of respondents conducted PAs independently without the assistance of clerical staff.^9^ Pharmacy technicians are a vital part of the team as they aid in submitting PAs and contacting insurers for status determinations. Working alongside pharmacy technicians, clinical pharmacists are able answer clinical questions and appeal denials with letters of medical necessity or peer-to-peer reviews. Our study found that pharmacy technicians were able to help obtain approval for 78% of all initial referrals without having to pursue an appeal. Even with 14% of referrals being for non-FDA-labeled indications, the clinical pharmacy team was able to successfully initiate or maintain patients on 98% of intended therapies. Adalimumab had a higher proportion of approvals (97%) while newer therapies such as ozanimod had a lower rate of approval (89%). Upadacitinib, which at the time of this analysis did not have FDA approval for UC, had a much lower rate of approval (76%). It is important to note that with 1800 referrals received, a total of 2064 PAs, appeals and patient assistance program applications were submitted. The 15% increase in submission volume compared with referral count showcases the required work performed by the clinical pharmacy team to start or maintain patients on intended therapy. This illustrates 1 aspect of support the clinic pharmacy team provides the IBD clinic.

This study described the scope of clinical interventions that clinical pharmacists could intervene on in IBD patients. A previous survey completed by gastroenterology and hepatology pharmacists found that majority of pharmacists practice in an outpatient setting with 71% having clinic responsibility (either seeing patients independently or in a multidisciplinary clinic).^9^ More than 60% of pharmacists develop therapeutic plans, order, and monitor labs, provide medication reconciliation as well as patient education. This study illustrates similar findings with clinical interventions predominately associated with therapy-related recommendations, patient education and lab monitoring for advanced IBD therapies.

Furthermore, studies have shown that pharmacist advanced IBD therapy involvement improved medication access and management when working alongside gastroenterologists.^4,11–14^ A multicenter study completed in Australia showed a correlation between IBD pharmacist interventions and improved patient adherence.^15^ Patients who were nonadherent at baseline experienced a decrease in nonadherence rates from 100% nonadherence to 44.4% nonadherence (*P* = .001) at 24 months.^15^ A recent retrospective study illustrated the success of pharmacists identifying and converting patients to biosimilars.^16^ In the study, pharmacists were able to successfully transition 97% of patients to an infliximab biosimilar.

As more pharmacists become integrated with IBD clinics it will be important to have a presence in clinic to establish themselves as part of the multidisciplinary team. This study demonstrates why pharmacists should be involved with the initiation and maintenance of all advanced IBD therapy and in clinic presence allows for optimal access to complete patient education, medication reconciliation as well as to develop and implement therapeutic plans. Not only initial medication implementation, but follow-up lab monitoring as well. The scope can even extend beyond advanced IBD therapy to population health, smoking cessation and a vaccination program.^17,18^ Although not measured in this analysis, the existence of the clinical pharmacy team also spares the physicians, nurses, and advanced practice providers from the duties of securing PAs, reauthorizations, and managing most of the appeals to payers.

With timely initiation being an important consideration for IBD patients, PAs and appeals have been shown to delay therapy initiation.^1,2,5^ With PA determinations taking at least 72 hours and appeal determinations taking at least 14 days, utilizing an IBD clinical pharmacy team allows for patients to start therapy a median of 11 days from initial referral request.^19^ With a 98% approval rate, regardless of 14% of referrals being for non-FDA-labeled indications and 15% of referrals requiring at least 1 level of appeal and over 2000 clinical interventions, the clinical pharmacy team is a crucial part of the IBD care team and a best practice in IBD centers.

Lastly, because pharmacists are performing clinical functions, it should be advocated that a collaborative practice agreement be in place. This allows providers to delegate duties to the pharmacist and allows for independent management and expansion of services. However, this must be assessed on a state level as each state has different laws and regulations around collaborative practice agreements.^20^

There were a few limitations to this analysis. Due to the retrospective nature of this study, there may have been documentation that was not captured, and the number of clinical interventions may have been underestimated. For example, routine follow-up assessments and side effect management strategies that take place within those follow-up activities were not captured as clinical interventions. Additionally, these routine follow-up activities are only completed in patients that fill their medication at UCM and performed as needed for patients filling through other specialty pharmacies. Being a single-center study, the data will also not reflect all patient populations, insurers, and provider prescribing patterns. This study did not include patients receiving infusions given different service lines are responsible for these referrals at our institution. Evaluating the comprehensive role of a clinical pharmacy team in the management of advanced IBD therapy infusions will need to be further evaluated in future studies.

## Conclusions

This is the first study to comprehensively evaluate the role of a clinical pharmacy team at a tertiary academic IBD center for self-administered advanced IBD therapies and the value added both clinically and operationally. Even with the high utilization of non-FDA-approved indications, the clinical pharmacy team illustrated the ability to obtain insurance approval for 98% patients. Not only obtaining insurance approval and ensuring affordability, the 2 IBD pharmacists made significant clinical contributions with respect to preventing lapse in therapy, patient education, therapy change management, and appropriate lab monitoring.

## Data Availability

Data not publicly available.
